# Impact of infection control educational activities on the rates and frequencies of percutaeous injuries (PIS) at a tertiary care hospital in Saudi Arabia

**DOI:** 10.1186/1753-6561-5-S6-P219

**Published:** 2011-06-29

**Authors:** HH Balkhy, K El Beltagy, A El-Saeed, M Sallah

**Affiliations:** 1 Infection Prevention and Control, Riyadh, Saudi Arabia; 2 KAMC, Riyadh, Saudi Arabia

## Introduction / objectives

To study the Impact of educational activities on the rates and frequencies of Percutaneous Injuries at a tertiary care hospital in Saudi Arabia.

## Methods

PIs Surveillance is a routine activity in King Abdulaziz Medical City in Riyadh using the Exposure Prevention Information Network (EPINet) data collection tool. On 2001 through 2003, we started educational activities of HCWs aiming at preventing PIs. This education included lectures and inservice training about the risk factors and unsafe practices contributing to PIs and how to avoid it. Data before the intervention (1997-2000) and after the intervention (2004 - 2008) were imported from our surveillance system and statistically analyzed.

## Results

Compared to the pre-interventional period**, **the overall rate of PIs during the post intervention period has dropped significantly (14% vs. 32.8% per 1000 HCWs). The rates among nurses and housekeepers showed a significant drop (15% vs. 37.6% for nurses and 10% vs. 34.5% for housekeepers). PIs frequencies in Emergency Department (ED) and Intensive Care Units (ICU) showed significant decrease (3.4% for both vs. 12.4 % & 13.7% respectively). Devices as needle on IV line, IV catheters, lancets and suture needles showed significant decrease in frequency. PIs frequencies occurring during device disassembly and devices left inappropriately showed significant decrease.

## Conclusion

The educational program appeared to have positive impact on reducing some categories of PIs including the overall rate, nurses and housekeepers job categories, ED and ICU, Needle on IV line, IV catheters, lancets and suture needles. However, other PIs categories did not change significantly.

## Disclosure of interest

None declared.

